# The Genome of a Thermo Tolerant, Pathogenic Albino *Aspergillus fumigatus*

**DOI:** 10.3389/fmicb.2018.01827

**Published:** 2018-08-14

**Authors:** Brian Couger, Tyler Weirick, André R. L. Damásio, Fernando Segato, Maria De Lourdes Teixeira De Moraes Polizeli, Ricardo S. C. de Almeida, Gustavo H. Goldman, Rolf A. Prade

**Affiliations:** ^1^Department of Microbiology and Molecular Genetics, Oklahoma State University, Stillwater, OK, United States; ^2^Faculdade de Filosofia, Ciências e Letras de Ribeirão Preto, São Paulo, Brazil; ^3^Laboratório Nacional de Ciência e Tecnologia do Bioetanol, Campinas São Paulo, Brazil; ^4^Departamento de Biotecnologia da Escola de Engenharia de Lorena, Universidade de São Paulo, São Paulo, Brazil; ^5^Faculdade de Ciências Farmacêuticas de Ribeirão Preto, São Paulo, Brazil

**Keywords:** *Aspergillus fumigatus*, *Aspergillus fumigatus* var. *niveus*, genome sequencing, aspergillosis, glycosyl hydrolases, secretome, pathogenesis

## Abstract

Biotechnologists are interested in thermo tolerant fungi to manufacture enzymes active and stable at high temperatures, because they provide improved catalytic efficiency, strengthen enzyme substrate interactions, accelerate substrate enzyme conversion rates, enhance mass transfer, lower substrate viscosity, lessen contamination risk and offer the potential for enzyme recycling. Members of the genus *Aspergillus* live a wide variety of lifestyles, some embrace GRAS status routinely employed in food processing while others such as *Aspergillus fumigatus* are human pathogens. *A. fumigatus* produces melanins, pyomelanin protects the fungus against reactive oxygen species and DHN melanin produced by the *pksP* gene cluster confers the gray-greenish color. *pksP* mutants are attenuated in virulence. Here we report on the genomic DNA sequence of a thermo tolerant albino *Aspergillus* isolated from rain forest composted floors. Unexpectedly, the nucleotide sequence was 95.7% identical to the reported by *Aspergillus fumigatus* Af293. Genome size and predicted gene models were also highly similar, however differences in DNA content and conservation were observed. The albino strain, classified as *Aspergillus fumigatus* var. *niveus*, had 160 gene models not present in *A. fumigatus* Af293 and *A. fumigatus* Af293 had 647 not found in the albino strain. Furthermore, the major pigment generating gene cluster *pksP* appeared to have undergone genomic rearrangements and a key tyrosinase present in many aspergilli was missing from the genome. Remarkably however, despite the lack of pigmentation *A. fumigatus* var. *niveus* killed neutropenic mice and survived macrophage engulfment at similar rates as *A. fumigatus* Af293.

## Introduction

The search for thermo tolerant organisms that manufacture enzymes active and stable at high temperatures has been investigated in fungi, (Berka et al., 2011; Brink et al., 2011; Houbraken et al., 2012; Morgenstern et al., 2012), bacteria (Chang and Yao, 2011; Mingardon et al., 2011; Wang et al., 2011; dos Santos et al., 2012; Bhalla et al., 2013; Sato and Atomi, 2013) and archaea (Cavicchioli et al., 2011; Wackett, 2011; Cadillo-Quiroz et al., 2012; Davidova et al., 2012). Raising the temperature accelerates hydrolytic rates, provides a robust conversion rate in addition to enhanced mass transfer, reduced substrate viscosity and the potential for enzyme recycling (Wojtczak et al., 1987; Haki and Rakshit, 2003; Viikari et al., 2007; Berka et al., 2011). Thus, thermo tolerant fungi represent a prospective genomic resource for thermostable enzyme discovery.

The genus *Aspergillus* groups a large number of species conveying a wide variety of lifestyles reciprocally beneficial and detrimental to humans (Gibbons and Rokas, 2013). Some species embrace GRAS (generally regarded as safe) status routinely employed for hundreds of years in food processing while others such as *Aspergillus fumigatus* are serious human pathogens causing aspergillosis (Latgé, 2001). Within the group of fungi that detrimentally affect humans, thermotolerance appears as a determining factor necessary to establish a pathogenic relationship (Casadevall, 2007). Nevertheless, humans have powerful innate immune mechanisms and pathogenic fungi possess the ability to withstand the negative effects of innate immune systems that include microbicidal peptides, oxidative bursts, phagocytic cells, and nutrient deprivation (Casadevall, 2007).

*A. fumigatus* Af293 produces at least two types of melanins, pyomelanin and dihydroxynaphthalene (DHN). Pyomelanin protects the fungus against reactive oxygen species, however mutants defective in pyomelanin production are not affected in virulence (Keller et al., 2011; Heinekamp et al., 2012). DHN melanin is produced by the *pksP* gene cluster and confers the gray-greenish color of conidia and mutants lacking a functional polyketide synthase (PksP) are attenuated in virulence (Pihet et al., 2009; Heinekamp et al., 2012; Bayry et al., 2014).

Here we report on the genome of a thermo tolerant albino *Aspergillus* isolated from a comprehensive screen on Brazilian rain forest composted floors. Unexpectedly, the nucleotide sequence was 95.7% identical to the reported for *Aspergillus fumigatus* Af293 (Nierman et al., 2005). Based on DNA sequence phylogenetic and morphotaxonomic criteria the fungus was named *Aspergillus fumigatus* var. *niveus* (previously *Aspergillus niveus*). Furthermore, genome size and the predicted gene models were also highly similar. However, some differences in gene model organization and species-specific unique DNA content was observed.

## Materials and methods

*Aspergillus nidulans* FGSC A1228 (*yA2, pabaA1*) and *Aspergillus fumigatus* Af293 was purchased from the Fungal Genetics Stock Center (FGSC, St Louis, MO) and *Aspergillus awamori* strain ATCC 22342 was a gift from the National Renewable Energy Laboratory (NREL, Golden, CO). *Aspergillus fumigatus* var. *niveus (*AFUMN) was isolated from a composted tropical forest-floor in Brazil, initially classified as *Aspergillus niveus* by the URM Mycology Collection, Department of Mycology of the Federal University of Pernambuco (Recife, PE Brazil) and is available for distribution.

### Media, strains, cultivation, and solutions

One liter of complete medium contained 50 ml of 20X Clutterbuck salts (Clutterbuck, 1992), 1 ml of 1000X vitamins, 1 ml of 1000X trace elements, 5 g of tryptone, 2.5 g of yeast extract and 10 g of glucose titrated at pH 6.5. Vegetative cultures and spore production were prepared by inoculation of conidia in minimal medium as described in Clutterbuck (1992) and Pontecorvo et al. (1953).

### Temperature-dependent vegetative growth rate assays

Single colony agar plate assays were generated by single-spot inoculation at the center of a complete medium agar plate with fresh conidia from *A. fumigatus* var. *niveus, A. fumigatus* Af293, *A. awamori* and *A. nidulans*. Plates were incubated at various temperatures, 30°, 37°, 45°, and 55°C and vegetative exponential growth rate determined by measuring the colony diameter (in millimeters) after 21 and 45 h of growth. Exponential vegetative growth rate was calculated within the 22nd and 45th hour time frame and rates reported as millimeters per hour.

### Genomic DNA sequencing, contig assembly, and phylogenetic trees

To extract high molecular weight genomic DNA (gDNA); spores were grown on liquid minimal medium on Petri dishes for no more than 24 h. Mycelial mats were harvested, washed with sterile water, and frozen with liquid nitrogen. Mycelia were lysed by addition of 1 ml of genomic extraction solution (1% SDS, 50 mM EDTA) to 200 mg of tissue, heated for 20 min. at 68°C, and separated by centrifugation. The supernatant was transferred to a new tube, 45 μl of 5 M potassium acetate added, incubated on ice 10–30 min and centrifuged. 400 μl of supernatant was transferred to a new tube containing 950 μl of 95% ethanol and the gDNA fished out with a glass hook, washed with 70% ethanol, and suspended in TE with 1 μg/ml RNAse.

Genomic sequencing was conducted using a combination of HiSeq (Illumina GAIIx, Sequensys, La Jolla, CA) 150 × 2 paired-end reads and Roche 454 FLX Titanium Sequencing (Creative Genomics, Port Jefferson Station, NY). Four micrograms of gDNA was used to generate libraries using the standard Illumina TruSeq protocol with an average sequencing insert size of ~500 bp. All sequencing generated reads were co-assembled with the short read De Bruijn graph assembly program velvet (Zerbino and Birney, 2008) using the following settings; kmer value of 47, minimum coverage of 5, Roche reads as “long” read type, and a minimum contig length of 300 bp. The resulting assembly contained 27.4 MB having an n50 of 125 kb (Table 1 and Table S1).

**Table 1:** Comparison of AFUMN genome topography data.

	**AFUMN**	***A fumigatus***
**GENOME**		
Genome sequenced size	27.3 Mb	29.4 Mb
G+C content	50.0%	49.9%
Gene Models	8,909	9,926
Mean GM length	1,670 bp	1,431 bp
Coding genome fraction	54.4%	50.1%
GMs with introns	78.2%	77.0%
GMs with unclear functions	3,105	3,288
**EXONS**		
Mean number per GM	2.9	2.8
Mean length of exon	514 bp	516 bp
G+C content	54.0%	54.0%
**INTRONS**		
Mean number per GM	1.9	1.8
Mean length	86 bp	112 bp
G+C content	46.2%	46.0%
**RNA**		
tRNAs	147	179

*Consult Table S1 for complete meta data statistics*.

Phylogenetic neighbor joining trees were analyzed with MEGA (Kumar et al., 2008; Tamura et al., 2011), and to support clades a bootstrap analysis was performed with 1000 replications. The DNA sequence data form aspergilli were from Varga et al. (2007) and *A. fumigatus* var. *niveus* from this study.

The organism sample (Biosample: SAMN02628958), taxonomy (Organism: JHOI01000000) and whole AFUMN genome DNA sequence (Bioproject PRJNA237468) along with all predicted gene models have been submitted to the National Center for Biotechnology Information (NCBI).

### Gene calling, gene model construction, and genome distance calculations

Gene calling was conducted using a combination of *ab initio* gene calling programs and transcript to genome alignments. The gene calling programs GlimmerHMM (Majoros et al., 2004) and Augustus (Stanke et al., 2008) were used for gene *ab initio* prediction with *A. fumigatus* Af293 mRNA training set models. All refseq *A. fumigatus* Af293 mRNA models were aligned to the *Aspergillus fumigatus* var. *niveus* genome, using Program to Assemble Spliced Assemblies, PASA (Haas et al., 2008) and final single locus-best prediction consensus models were created using EVidenceModeler, EVM (Haas et al., 2008). This gene model construction pipeline resulted in 8,909 gene models (Table 1) with an average predicted protein length of 502 amino acids, 99.4% of them having a BLAST first hit *e*-value of E-5 or less, using the NCBI non-redundant protein sequence database (nr).

Genome to genome distances were calculated by “*in silico*” DDH (DNA-DNA hybridization) with our AFUMN genome and several of sequenced Aspergilli using the algorithm described by Auch and cols (Auch et al., 2010; Meier-Kolthoff et al., 2013).

### Functional annotation, secretome analysis, and comparative genomics

Protein sequences from the final gene models were functionally annotated using a combination of homology prediction, domain prediction, cellular localization prediction, and comparative genomic analysis. Closest homologs to all predicted protein sequences were identified using the NCBI BLAST+ (Camacho et al., 2009) program using the *nr* database. Domain predictions for all protein models were achieved by using the HMMER (Eddy, 2011) software packages HMMSCAN module having the PFAM 26.0 as the reference domain database (Punta et al., 2012). All domains with an *e*-value of E-5 or less were considered valid for domain classification. Domains present in CAZy (Carbohydrate-Active EnZymes database) (Cantarel et al., 2009; Yin et al., 2012; Levasseur et al., 2013) as well as the *A. fumigatus var. niveus* genome were used for carbohydrate metabolism classification. Putative secreted proteins were identified using the eukaryotic signal peptide prediction software SignalP 4.0 (Petersen et al., 2011). Comparative genomic analysis within the *A. fumigatus* group (*A. fumigatus* var. *niveus, A. fumigatus* Af293, *A. fumigatus* A1163, *N. fischeri*, and *A. clavatus*) was performed using a combination of bi-directional and HMMSCAN/PFAM comparisons. The genomes/proteomes of the *A. fumigatus* group were obtained from the Broad Institute *Aspergillus* Comparative Sequencing Project (Broad Institute of Harvard and MIT) and the A1163 genome from the NCBI bioproject id PRJNA18733.

### Murine model of pulmonary aspergillosis

BALB/c mice (body weight, 20–22 g) were housed in vented cages containing 5 animals. Mice were immunosuppressed as described in Dinamarco et al. (2012). *A. fumigatus* Af293 or *A. fumigatus* var. *niveus* spores used for inoculation were grown on *Aspergillus* complete medium (Clutterbuck, 1992) for 2 days prior to infection. Conidia were freshly harvested in PBS and filtered through a Miracloth (Calbiochem). Conidial suspensions were spun for 5 min at 3,000 × G, washed three times with PBS counted using a hemocytometer, and resuspended at a concentration of 2.5 × 10^6^ conidia/ml. Counts of viable inoculants were determined by following colony counts on serial dilutions plated on complete medium and incubations at 37°C for 2 days. Mice were anesthetized by halothane inhalation and infected by intranasal instillation of 5.0 × 10^4^ conidia in 20 μl of PBS. As a negative control, a group of 5 mice received PBS only. Mice were weighed every 24 h from the day of infection and visually inspected twice daily. In most cases, the endpoint for survival experimentation was identified when a 20% reduction in body weight was recorded, at which time the mice were sacrificed. The statistical significance of comparative survival values was calculated using log rank analysis and the PRISM statistical analysis package. Animal handling employed in this study were approved by the local ethics committee of the University of São Paulo, Campus of Ribeirão Preto (PN 08.1.1277.53.6;) and follows articles 8 and 14 of the Declaration of Animal Rights ratified by UNESCO in January 27, 1978.

### Conidial killing by alveolar macrophages

To evaluate conidial killing by murine alveolar macrophages phagocytic cells were harvested as described in Dinamarco et al. (2012). Briefly, in a 96-well plate, 5 × 10^4^ murine macrophages were added to 200 μl of RPMI 1640, 10% FCS per well and incubated at 37°C with 5% CO_2_ for 5 h. After 1 h 2.5 × 10^5^ conidia (5 conidia per macrophage) were added. A positive control contained medium and spores without macrophages. Triplicate wells were assayed for each strain (*A. fumigatus* var. niveus and *A. fumigatus* Af293) with and without macrophages. After incubation, the micro titer dish plate was centrifuged at 3,500 rpm for 10 min, supernatants removed, and 100 μl of 1% Triton X-100 was added. After a 10 min room temperature incubation, samples were removed from wells and serially diluted in sterile water and plated on *A. fumigatus* complete medium (Mech et al., 2011), incubated at 37°C for 2 days. The percentage of conidial killing was calculated by comparing CFU numbers from samples incubated with macrophages and without macrophages. Experiments were repeated three times.

## Results and discussion

**Figure 1:**
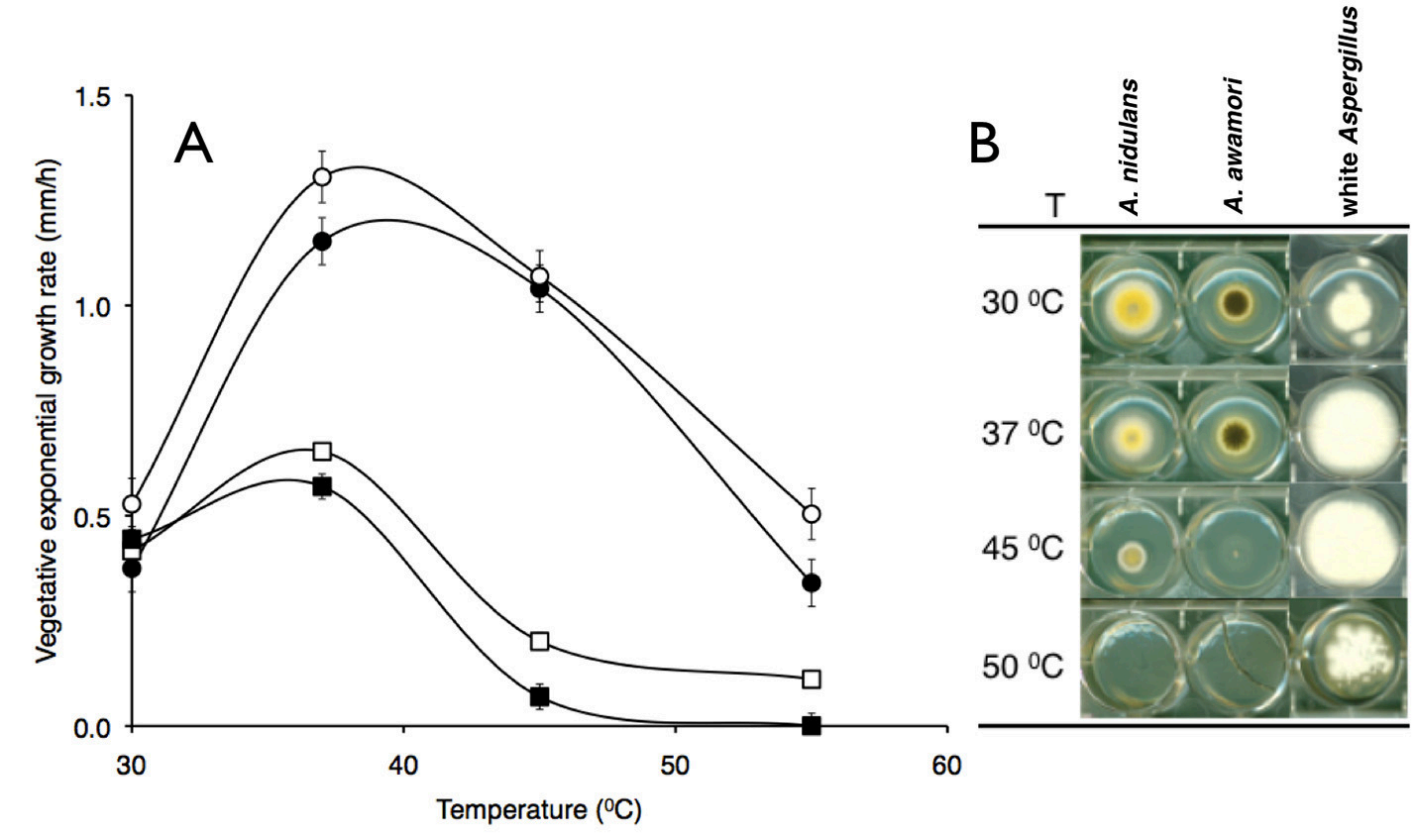
Temperature-dependent vegetative exponential growth rate of aspergilla. Colonies of four aspergilli were single-colony inoculated onto complete medium and radial growth rates (mm/h) measured between the 22nd and 45th hour of continuous vegetative growth at indicated temperatures. **(A)** *A. fumigatus* Af293 and *A. fumigatus* var. *niveus* (open and closed circles), *A. nidulans* and *A. awamori* (open and closed squares) vegetative exponential growth rates (reported as millimeters per hour). **(B)** Vegetative colony morphology of aspergilli grown at various temperatures labeled as shown.

A white spored *Aspergillus* which produced colonies devoid of pigmentation was identified as a thermo tolerant filamentous fungus living on forest floor composting environments in Brazil. Figure 1A compares temperature-dependent vegetative exponential growth rates of this white *Aspergillus* isolate with three well-known aspergilli. Comparably, the white *Aspergillus* and *A. fumigatus* Af293 showed similar high temperature profiles while in contrast other aspergilli, *A. awamori* and *A. nidulans* showed a lower-range temperature profile (Figure 1A). The white *Aspergillus* strain appeared to be albino devoid of any type of pigmentation (Figure 1B), white mycelium and aerial hyphae, white conidiospores and no evidence of pigmentation leaked on the back of an agar plate (data not shown).

### The white thermophilic *aspergillus* is *aspergillus fumigatus* var. *niveus*

**Figure 2:**
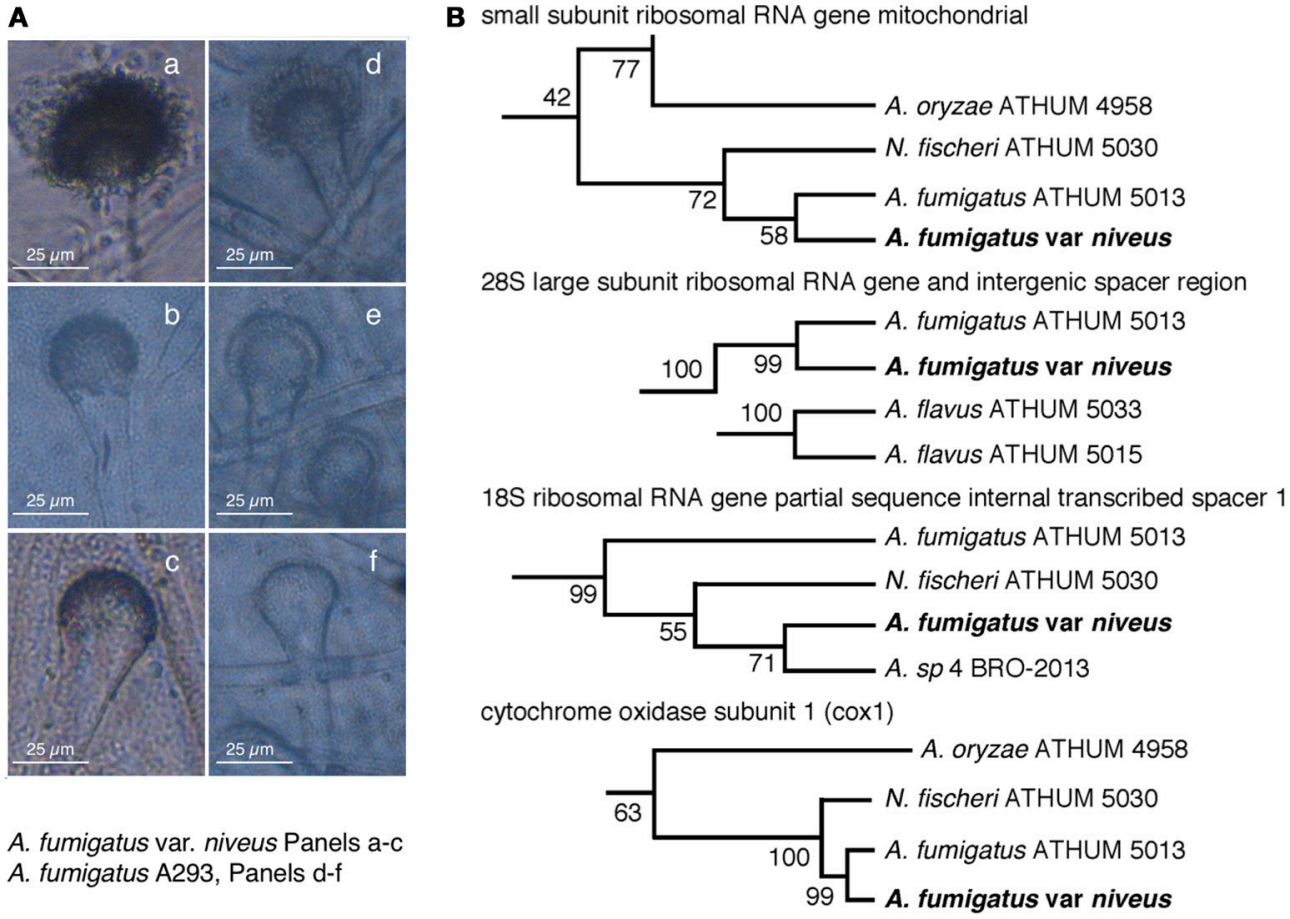
Taxonomic identification of an albino *Aspergillus* as *Aspergillus fumigatus* var. *niveus* (AFUMN). **(A)** Morphotaxonomy of aspergilli conidiophore vesicle shape. **(a**–**c)** and **(d**–**f)** show the albino *Aspergillus* and *Aspergillus fumigatus* Af293 conidiophore vesicles, respectively. **(B)** Ribosomal RNA neighbor-joining phylotaxonomy of small 18S and large 28S subunit internal spacer sequences cluster the albino *Aspergillus* isolate with *Aspergillus fumigatus* ATHUM 5013 and *Neosartoria fisheri* ATHUM 5030. Only a small section of a complete phylogenetic tree is shown. Full trees with bootstrap values can be found in Figure S1.

The high degree of gDNA sequence similarity (see below) of the albino *Aspergillus* with *A. fumigatus* isolates and related species such as *Neosartorya fischeri*, lead us to compare in Figure 2A the morphology of condiophore vesicles and uniseriate phialide that were almost identical between strains. Thus we further constructed ribosomal RNA neighbor-joining phylotaxonomy of small 18S and large 28S subunit internal spacer sequences (Figure 2B) including 26 aspergilli and the albino *Aspergillus* which revealed consistent clustering with *A. fumigatus* ATHUM5013, *N. fischeri* ATHUM 5030, and *A. felis* 4 BRO-2013 suggesting that our strain was a member of the *A. fumigatus* family (Varga et al., 2007; Barrs et al., 2013). The DNA sequence data form aspergilli were from (Varga et al., 2007) and *A. fumigatus* var. *niveus* from this study. Full trees with bootstrap values can be found in Figure S1.

### Genome content of *aspergillus fumigatus* var. *niveus*

The genomic DNA (gDNA) sequencing of AFUMN resulted in 27.3 Mb of finished and validated gDNA distributed in 671 scaffolds (Table 1 and Table S1). Initial “*ab initio*” *A. fumigatus* Af293 trained gene calling (GlimmerHMM/Augustus) resulted in 18,578 gene models while *A. fumigatus* Af293 transcripts mapping to the AFUMN genome via GMAP and PASA produced 24,716 GMAP alignments and 7,293 PASA transcript assemblies, respectively resulting in a final set of consolidated 8,909 EvidenceModeler gene models (GM) (see section Materials and Methods and Table S1). The 8,909 validated GMs covered 14.8 Mb of the 27.3 Mb genomic sequence, suggested an average gene density of one gene every 3.05 kb or 1 coding base pair for every 1.8 genomic base pair.

The gDNA sequence of AFUMN was similar to *A. fumigatus* Af293 as well as genome size, 27.3 and 29.4 Mb; G+C% content, 49.9 and 50.0; mean GM length, 1,431 and 1,670 bp; genome coverage with exons, 50.1 and 54.4% and the number of predicted GMs, 9,926 and 8,909, respectively (Table 1). *A. fumigatus* Af293 data were from Nierman's group (Nierman et al., 2005). The 8,909 predicted number of GMs and gene density of one gene per 1.6 kb was in agreement with the predictions based on genome size and gene density of eukaryotic organisms (Kupfer et al., 1997). Furthermore, using a HMMSCAN domain search against the Core Eukaryotic Gene data set (CEGMA), 447 of the 458 conserved CEGMA COG Eukaryotic Core Genes were identified in AFUMN suggesting a completion rate of 97.5% (Parra et al., 2007, 2009).

### Nearly all *a. fumigatus* var. *niveus* GMs are identical to *a. fumigatus*

Genome sequence comparisons among three aspergilli, *A. nidulans, A. fumigatus* Af293 and *A. oryzae* indicated through three-way ortholog comparisons that they retained 66–70% amino acid identity (Galagan et al., 2005). The AFUMN genomic DNA sequence was 95.7% identical to *A. fumigatus* Af293 (Table 2 and Table S2). Furthermore, comparisons of GM orthologs between AFUMN and *A. fumigatus* (Af293 and A1163) and/or *N. fischeri* revealed 8,909 GMs at 98.3% identity levels (Table 2). The identity among GMs was highest in orthologs that encoded gene products with known functions (99.5%) and slightly less conserved (96%) among gene models with unclear functions (unknowns, hypothetical, conserved protein etc.). Table 2 showed that 1.7% (151 GMs) of all analyzed GMs had similarity to GMs derived from species other than *A. fumigatus*.

**Table 2:** Nearly all AFUMN GMs are identical to *A. fumigatus*.

**Gene models (GM)**	**AFUMN**	***A. fumigatus and N. fischeri***		**Other fungi**	
		**GM**	**%**	**GM**	**%**
Total	8,909	8,758	98.3	151	1.7
Clear biochemical function	5,845	5,818	99.5	27	0.5
Unclear biochemical function	3,064	2,940	96.0	124	4.0

*Consult Table S2 for itemized list of AFUMN gene models*.

DNA sequence identity rates reported earlier (95.7%) were calculated by arbitrary choosing of DNA fragments and direct DNA-DNA comparisons. A more pragmatic approach of measuring DNA sequence similarity would be “*in silico*” DNA-DNA hybridization on entire genomes (DDH) and enable estimation of the overall similarity between genomes (Auch et al., 2010). Thus, we compared the AFUMN genome with several other aspergilli and found that AFUMN was 97.8, 97.1, 66.1, and 13.2 DDH identical to *A. fumigatus* A1163, *A. fumigatus* Af293, *N. fischeri* and *A. acidus* CBS 106, respectively (Auch et al., 2010).

### Some GMs are *A. fumigatus* var. *niveus* specific

From the global GM inventory (Tables 1, 2) it appeared that AFUMN was *A. fumigatus* Af293 with the exception that pigments were not produced, the mycelium was colorless with no leakage of any kind of pigment (often observed in aspergilli that produce colored conidia) and white uniseriate conidiospores (Figure 1B). Thus, we searched the AFUMN genomic sequence for GMs that were not present in any of the sequenced *A. fumigatus* genomes. We found 160 GMs not present in *A. fumigatus* (Table 3), whereas 78% of them were unknown or uncharacterized and 22% could be matched to a biochemical function (Table 3 and Table S3). From the 160 GMs that were not found in *A. fumigatus*, 66 (41%) were like other aspergilli, 84 (53%) resembling other fungi, and only 10 (6%) GMs were not like any other GM present in GenBank, NCBI or EMBL (Table 4 and Table S3).

**Table 3:** AFUMN unique GM inventory.

***A. niveus* unique GMs**	**GM**					
	**All**		**No Hit^a^**		**Hit^a^**	**(< e-20)**
GMs not in *A. fumigatus*	160	–	60	38%	100	63%
Housekeeping (HSK)	26	16%	0	–	26	16%
Non housekeeping (nHSK)	9	6%	0	–	9	6%
Unknown function	125	78%	60	45%	65	52%

a No hit everything with E-value p = >e-20 in GB, EMBL, or NCBI. *Consult Table S3 for a itemized list of all AFUMN unique genes*.

**Table 4:** Half of the AFUMN unique GMs were from other non aspergillii fungi.

**Closest ortholog not in *A. fumigatus***	**HSK**		**nHSK**		**Unknown function**				**All unique**	
					**Hit^a^** **(**<**e-20)**		**No hit^a^**		
	**GM**	**%**	**GM**	**%**	**GM**	**%**	**GM**	**%**	**GM**	**%**
Homologous to aspergillii	11	42	4	44	41	63	10	17	66	41
Homologous to other fungi	7	27	3	33	24	37	50	83	84	53
Not homologous to fungi	8	31	2	22	0	–	0	–	10	6
All GMs	26	–	9	–	65	–	60	–	160	–
	35(22%)				125(78%)			

a *No hit everything with E-value p = >e-20 in GB, EMBL, or NCBI*.

*Consult Table S3 for a itemized list of all AFUMN unique genes*.

From the 125 GMs annotated with an unknown function unique to AFUMN, 65 had high scoring homologs (*E*-value ≤ e-20) like other fungi and 60 GMs with lower *E*-values linked to bacteria, metazoan or vertebrates (Table 4). Eighty-seven GMs were like fungi, 27 resembled bacteria, 5 appeared to be metazoan and 6 were of apparent vertebrate ancestry (Table S3).

### Not all *A. fumigatus* GMs are present in *A. fumigatus* var. *niveus*

Since a small fraction (160) of AFUMN GMs could not be matched to any of the sequenced *A. fumigatus* strains, we reasoned that conversely, there may be *A. fumigatus* GMs that could not be matched to AFUMN (Table 5). Surprisingly, we found 745.5 kb of DNA sequences missing in AFUMN which accounted for 317, 379, and 664 GMs from *A. fumigatus* Af293, *A. fumigatus* A1163, and *N. fischeri*, respectively (Table 5 and Table S4). However, in *A. fumigatus* Af293, A1163, and *N. fischeri* there were only 33, 27, and 40 GMs encoding known functions, respectively while the clear majority (average of 92%), were classified under putative, unknown, hypothetical or uncharacterized predicted protein. Currently, we do not understand why *A. fumigatus* Af293 has so many unique unknown, hypothetical or putative GMs.

**Table 5:** GMs in *A. fumigatus/fischeri* but not in AFUMN.

**Related species**	**All**	**Function**			
		**Known**	**%**	**Unknown**	**%**
*A. fumigatus A293*	317	33	10.4	284	89.6
*A. fumigatus A1163*	379	27	7.1	352	92.9
*Neosartorya fischeri*	664	40	6.0	624	94.0

*Consult Table S4 for a itemized list of GMs*.

**Figure 3:**
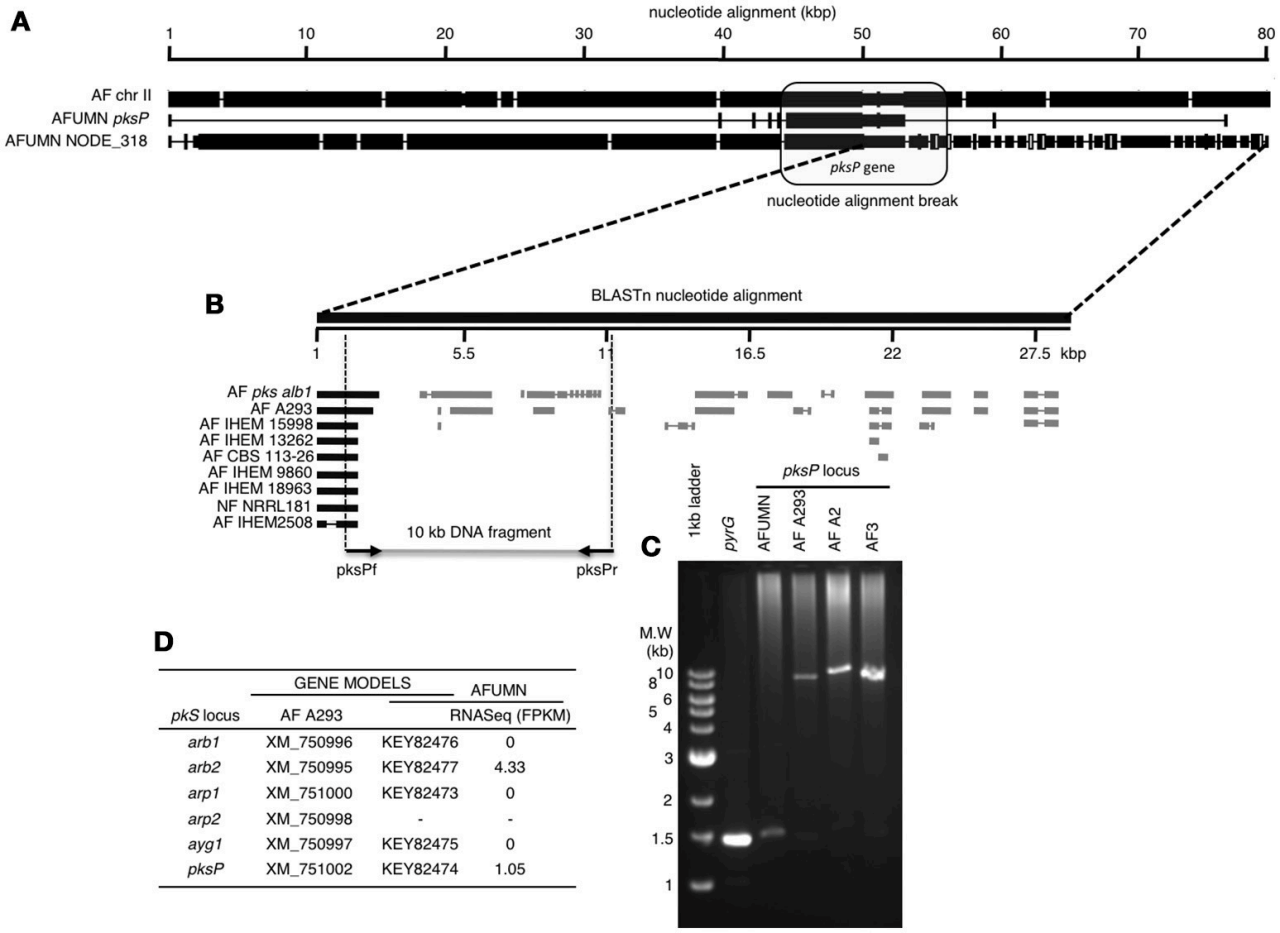
Nucleotide alignment of *A. fumigatus* var. *niveus* contig NODE_318, the *pksP* cluster and the corresponding region of *A. fumigatus* Af293 chromosome II. **(A)** In *A. fumigatus* var. *niveus* the *pksP* cluster is interrupted by non-*Aspergillus* DNA sequences. Closed and open boxes indicate nucleotide agreement and disagreement respectively, while interruptions indicate gaps (or deletions). In the initial 40 kb of the alignment there are 11 base-pair changes (deletion, insertion or base-pair change) while after the 50 kb mark the DNA sequence changes completely suggesting insertion of a foreign DNA or translocation. **(B)** Alignment of NCBI-nr fungal DNA with the conflicting terminal region of *A. fumigatus* var. *niveus pksP* cluster. The 27.5 kb DNA sequence initiating at the breakpoint in **(A)** (internal to the *pksP* cluster) was BLASTn aligned with all NCBI fungal DNA sequences and matches indicated by closed boxes (*pksP*) or gray boxes when aligning to other non-chromosome II DNA sequences. Empty spaces indicate DNA sequences not preset in NCBI-nr database. **(C)** PCR amplification of a 10 kb genomic DNA region surrounding the homology break in the *pksP* alignment in *A. fumigatus* var. *niveus* (AFUMN) and three *Aspergillus fumigatus* (AF293, AFA2, and AF3) strains. **(D)** The *A. fumigatus* (A293) *pksP* cluster genes contains six genes (*arb1, arb2, arp1, arp2, ayg1*, and *pksP*) one was not found in AFUMN four appear not to be transcribed in AFUMN and two showed baseline transcription levels.

Furthermore, a detailed analysis of the AFUMN *pksP* gene cluster region, the major locus responsible for conidial pigmentation (Sugui et al., 2007; Pihet et al., 2009; Heinekamp et al., 2012; Bayry et al., 2014), showed a clear break in synteny (Figure 3A) while compared to *A. fumigatus* Af293 and six other related isolates by means of recruitment of a new and unknown (not from *Aspergillus*) segment of DNA (Figure 3B). The DNA region that interrupts the *pksP* cluster is at least 28 kb in size, speckled throughout with DNA fragments of fungal origin (Figure 3B) and interspersed with DNA sequences of unknown origin (Figure 3B).

To discard a bioinformatics generated DNA assembly artifact in this region of AFUMN *pksP* region, we designed a pair of *A. fumigatus* primers that amplify a 10 kb DNA fragment, surrounding the synteny break region (Figure 3C). When *A. fumigatus* genomic DNA (three different strains) was used as a template for PCR amplifications a 10 kb DNA fragment was generated, however when *A. fumigatus* var *niveus* genomic DNA was used no PCR product was observed.

Finally, of the six *Aspergillus fumigatus pksP* cluster genes one was not found in AFUMN four appear not to be transcribed in AFUMN and two showed baseline transcription levels (Figure 3D).

In the case of *pksP*, this DNA sequence variation explained the lack of pigmentation of AFUMN, nonetheless also pointed to a more general mechanism to evolutionarily change based on the acquisition of foreign DNA (horizontal transfer), which could happen at a much faster rate than mutations on ribosomal genes.

Nevertheless, examination of genes not found in AFUMN but present in *A. fumigatus* Af293 and *A. clavatus* (Table S4), we found a GM encoding a pigment producing tyrosinase that was consistently missing in AFUMN but always present in *A. fumigatus* Af293 (XP_748428.1), *A. fumigatus* A1163 (EDP53552.1), *N. fischeri* (XP_001267634.1) and *A. clavatus* (three genes, XP_001276726.1, XP_001272230.1 and XP_001273482.1).

### Secretome inventory

Proteins secreted to the medium or anchored to the membrane architecture contain signal peptides that may perhaps be identified through bioinformatics (see section Materials and Methods). Table 6 and Table S5 reports global GMs with signal peptides grouped into their possible destination—membrane anchoring (9.5%), scaffolding architecture (3%), molecular transport (1.3%), and non-anchored proteins associated with environmental nutritional adaptations (47.3%). For about 38% of all putative GMs we were unable to assign a biochemical function. The identification of 749 predicted secreted proteins in AFUMN was in excellent agreement with other filamentous fungi such as *Penicillium chrysogenum, A. nidulans, Aspergillus niger, N. fischeri, Neurospora crassa, Ustilago maydis*, and *Trichoderma reesei*, which reported similar secretomes with 750–850 proteins (Lowe and Howlett, 2012).

**Table 6:** AFUMN secretome GM inventory.

**Predicted gene function category**	**GMs**	**Fraction %**
Environmental and nutritional adaptation	354	47.3
Structure scaffolding and architecture	22	2.9
Membrane anchoring and secretion	71	9.5
Molecular transport	10	1.3
Others	7	0.9
Unknown function	285	38.1
Gene models with secretion peptides	749	

*Consult Table S5 for a itemized list of GMs*.

### Glycosyl hydrolase inventory

Our initial interest in AFUMN was to identify and study GMs that encode enzymes that degrade plant cell wall polysaccharides. The Carbohydrate-Active enZYmes (CAZy) database compiles and assigns into families glycosyl hydrolases (GH), and other enzymes such as polysaccharide lyases (PL) and carbohydrate esterases (CE), according to a classification system based on amino acid sequence similarity, secondary and tertiary fold conservation, developed by Henrissat and coworkers (Henrissat and Bairoch, 1993; Levasseur et al., 2013). Table 7 describes all glycosyl hydrolase GMs found in AFUMN (274 GMs) and *A. fumigatus* Af293 (280 GMs). Both fungi had representatives in 65 of the 132 GH families and according to Jovanovic et al. (2009) from 114 (now 132) GH families only 22 were critical for biomass decomposition and 20 populated with genes from filamentous fungi (Jovanovic et al., 2009). Both *A. fumigatus* Af293 and AFUMN populate 18 of the 22 critical CAZy GH families (shaded boxes), whereas families GH 26, 44, 45, and 48 were not represented (Table 7). GH26 clustered β-mannanases (EC 3.2.1.78) and β-1,3-xylanases (EC 3.2.1.32) present in other aspergilli. However, families GH44, 45 and 48 which grouped endoglucanases and xyloglucanases, endoglucanases and reducing end acting cellobiohydrolases, respectively were not represented in aspergillii except for one GH45 endoglucanase from *A. nidulans*. In all there were only six CAZy differences between AFUMN (274 CAZy gene models) and *A. fumigatus* Af293 (280 CAZy gene models). AFUMN had 8 additional and 11 missing CAZy GM copies distributed over 6 and 8 GH families, respectively.

**Table 7:** CAZy glycosyl hydrolase (GH) Inventory of AFUMN and *A. fumigalus*.

	**0**	**1**	**2**	**3**	**4**	**5**	**6**	**7**	**8**	**9**
GH0_		5/5	6/6	17/17	3/3	12/13	1/1	5/5	1/1	1/1
GH1_	5/4	3/3	4/4	11/12		5/5	13/14	4/5	16/18	
GH2_	2/2			1/1		3/3		5/5	13/12	
GH3_	1/1	7/7	4/4	1/1		5/5	3/3	1/1	1/1	2/2
GH4_				18/18				5/5		
GH5_		2/2		1/1	1/1	6/7				
GH6_		AA9	2/2	2/2		1/1		1/1		
GH7_		4/8	7/7		3/3	4/4	8/8		6/5	
GH8_		1/1							2/2	1/1
GH9_			7/5	3/3		2/2				
GH10_						3/3			0/1	12/12
GH11_	1/0				1/1	1/1		1/0		
GH12_						1/1		1/1	1/2	
GH13_		2/2	2/2			Total CAZy GH GMs			274/280	

*Gene model count of AFUMN (274)/A. fumigatus Af293 (280) of CAZy glycosyl hydrolase orthologs. Light shaded boxes indicate GH families critical for biomass decomposition and dark shaded boxes indicates GH families with differences between AFUMN and A. fumigatus Af293*.

### *A*. *fumigatus* var. *niveus* is pathogenic as *a. fumigatus* AF293

The AFUMN genomic DNA sequence was very similar to *A. fumigatus*, both shared the vast majority of gene models even though AFUMN had some GMs that were not present in *A. fumigatus* and *A. fumigatus* did have unique GMs as well. There are many studies in *A. fumigatus* Af293 that indicate that pigmentation is a key factor involved in pathogenesis, specifically as auxiliary in protecting against the oxidative attack of macrophages and in inhibiting the acidification of the phagolysosome after conidial uptake (Jahn et al., 2002; Keller et al., 2011; Morton et al., 2011; Heinekamp et al., 2012). Thus, we rationalized whether AFUMN could be as pathogenic as *A. fumigatus* Af293 even though AFUMN appeared to be an albino version of *A. fumigatus* with no detectable pigmentation of hyphae and spores (Figure 1B).

**Figure 4:**
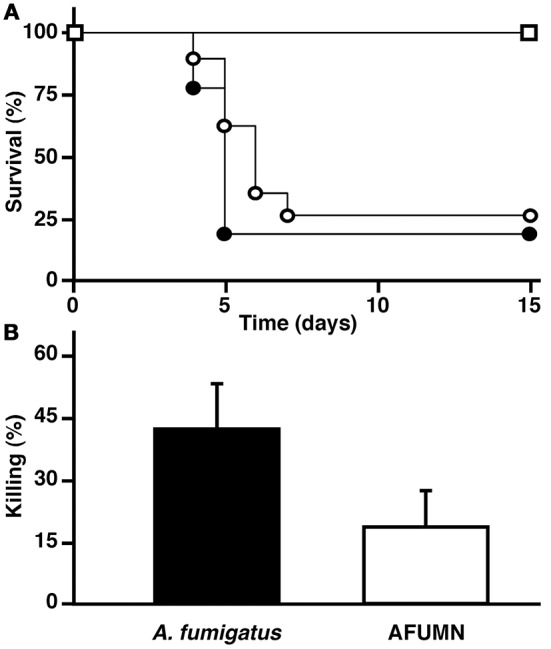
*A. fumigatus* var. *niveus* kills neutropenic mice and survives macrophage phagocytosis just like *A. fumigatus* Af293. **(A)** Comparison of *A. fumigatus* var. *niveus* (open circles) and *A. fumigatus* Af293 (closed circles) survival in a neutropenic murine model of pulmonary aspergillosis. BALB/c neutropenic mice were inoculated through intranasal instillation with of 5.0 × 10^4^ conidia and body-weight measured every 24 h and after reaching a 20% reduction, mice were sacrificed and statistical significance of comparative survival values calculated using log rank analysis and the PRISM statistical analysis package. **(B)** Conidial killing by murine alveolar macrophages assay. To evaluate conidial killing by murine alveolar macrophages, phagocytic cells were harvested and incubated with *A. fumigatus* var. *niveus* or *A. fumigatus* Af293 conidia (5 conidia per macrophage) and surviving conidia determined through CFU counting during serial dilutions.

To determine precise pathogenicity effects of AFUMN, we carried out two experiments; a neutropenic murine model of invasive pulmonary aspergillosis (Dannaoui et al., 1999; Sheppard et al., 2004; Steinbach et al., 2004; Clemons and Stevens, 2005; Seyedmousavi et al., 2011; Dinamarco et al., 2012; Heinekamp et al., 2012) and a conidial killing (or survival) by alveolar macrophage aggression. Intranasal delivery of AFUMN conidia killed neutropenic mice just as well as *A. fumigatus* Af293 conidia (Figure 4A). Moreover, AFUMN conidia can survive alveolar macrophage attacks at similar levels than *A. fumigatus* Af293 spores (Figure 4B), suggesting that AFUMN was just as pathogenic as *A. fumigatus* Af293. Elsewhere, pulmonary infection of a patient after allogeneic hematopoietic stem cell transplantation with *Aspergillus niveus* (Auberger et al., 2008) and invasive aspergillosis of humans, dogs and cats by *Aspergillus felis* (Barrs et al., 2013) has also been reported. The virulence of AFUMN in the immunocompetent and non-neutropenic models of pulmonary aspergillosis remains to be determined.

Both, AFUMN and *A. fumigatus* strains were exceptionally closely related in their DNA sequence and common habitats including infection of humans. In aspergilli, species differentiation determinants most likely are not the result of sequence variegation of single nucleotide mutations accumulated over time but rather the gain by one species or loss by another of entire DNA segments (horizontal gene transfer). AFUMN had 160 gene models that were not present in *A. fumigatus* and *A. fumigatus* had 647 gene models, 575 of which remain as putative or unknowns, were not found in AFUMN.

Finally, morphological trait classification (similar vegetative structures, asexual conidiophores, killing of neutropenic mice, macrophage engulfment survival) and phylogenetic DNA sequencing suggest that both fungi are the same species, however based on their unique DNA segments they may be different.

There are some striking similar cases among aspergilli. For example the genome size, DNA sequence and predicted gene models are also almost identical between *Aspergillus flavus* and *Aspergillus oryzae*, the former a plant pathogen producing the deadly aflatoxin and the latter harmless widely recognized as GRAS and employed in the food industry (Payne et al., 2006; Chang and Ehrlich, 2010). Cryptic speciation and recombination between these two aspergilli led to the conclusion long before whole genome sequencing became available that *A. oryzae* was a domesticated ecotype of wild *A. flavus* (Geiser et al., 1998; Gibbons et al., 2012). Remarkably, sequencing of Coccolithophores, the *Emiliania huxleyi* reference genome strain CCMP1516 and 13 additional isolates of marine phytoplankton, revealed a pan genome arrangement where a core set of common genes was appended and distributed variably among different isolates (Read et al., 2013). Other examples of horizontal gene transfer in aspergilli are well known such as fragmentation of an aflatoxin-like gene cluster in a forest pathogen (Bradshaw et al., 2013) and horizontal transfer and death of a fungal secondary metabolic gene cluster (Campbell et al., 2012).

## Conclusions

The finished genomes for the two thermo tolerant fungi, *A. fumigatus* and AFUMN, may serve not only as a reference genome for study on genome evolution and speciation, but may also support comparative analysis of thermophilic gene models useful in biotechnological applications. The differences between *A. fumigatus* var. *niveus* and *A. fumigatus* are not detectable at the DNA sequence level but in the acquisition (or loss) of specific DNA segments uniquely present in each one of the two species. Thus, we conclude that *A. fumigatus* var. *niveus* and *A. fumigatus* are the same species based on phylogenetic ancestry classification standards however are sufficiently different based on their unique set of DNA segments.

## Author contributions

GG, RP, MP, FS, and AD wrote the final version of the article. AD, FS, and RP performed all experiments, such as isolation of genomic DNA for sequencing, physiological properties determination, taxonomic identification and neutropenic mice survival curves and alveolar macrophage assays. BC and TW performed genome reconstructions and all other bioinformatics tasks. GG and RA supervised clinical pathology experiments and natural survival properties, respectively.

### Conflict of interest statement

The authors declare that the research was conducted in the absence of any commercial or financial relationships that could be construed as a potential conflict of interest.
