# Supplementary material for: The Genome of a Thermo Tolerant, Pathogenic Albino Aspergillus fumigatus
Source: Front Microbiol. 2018 Aug 14;9:1827. doi: 10.3389/fmicb.2018.01827 (PMC6102483; doi:10.3389/fmicb.2018.01827)
Supplement: Supplementary file 1 [file Table_1.pdf]

Figure S1 Expanded neighbor-joining phytotaxonomic trees and identification of the albino *Aspergillus fumigatus* var. *niveus* (AFUMN)

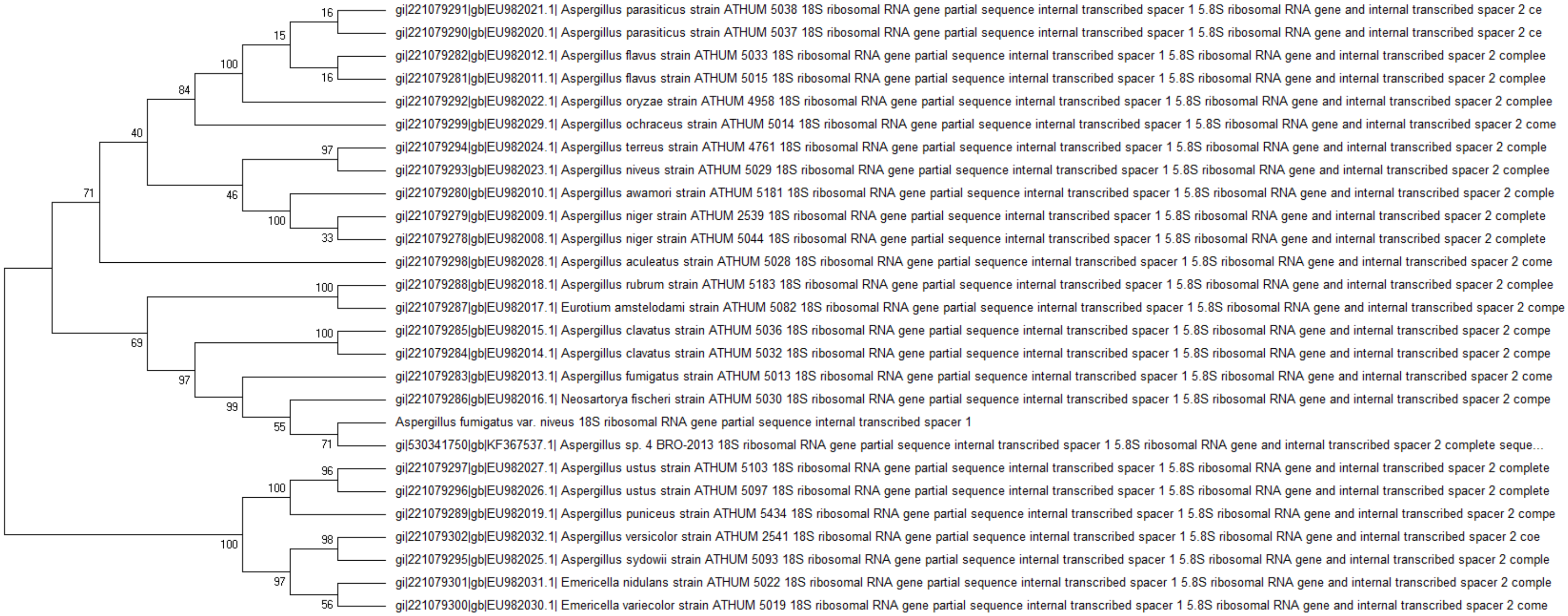

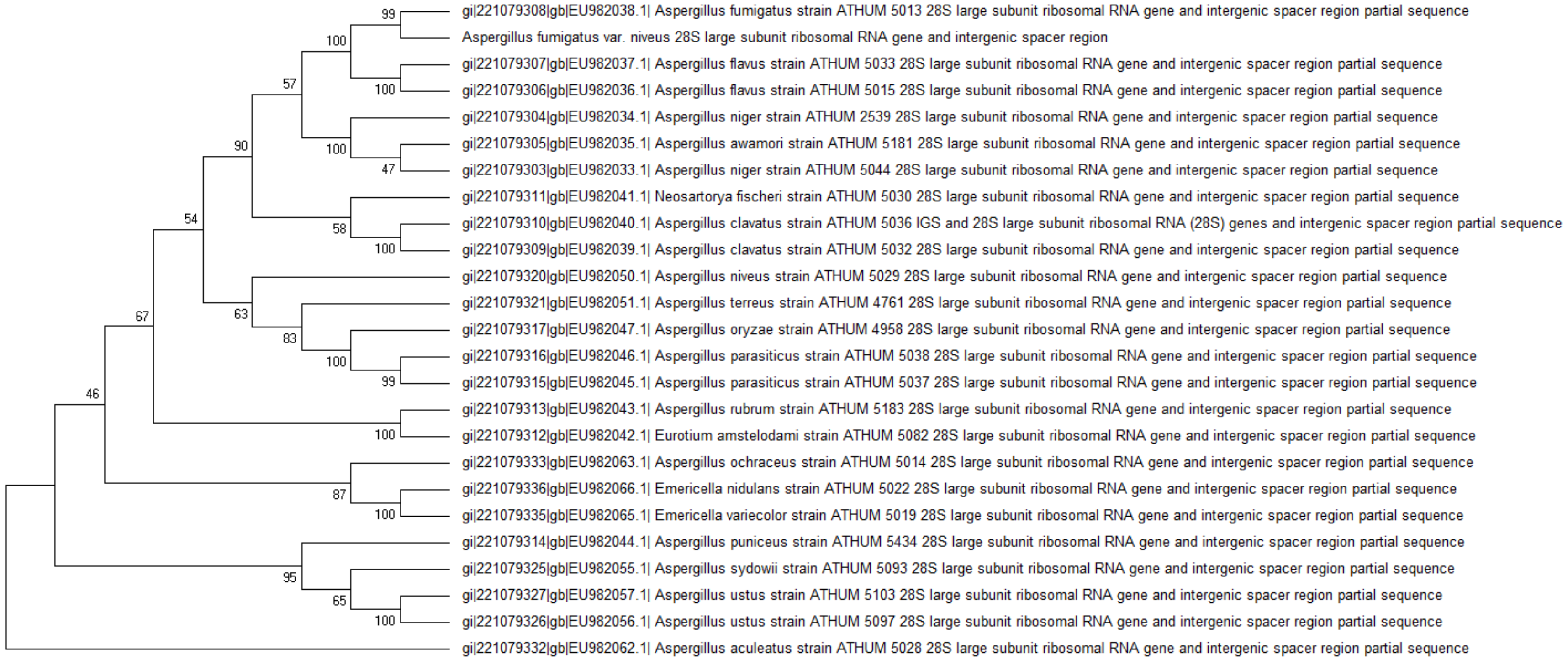
gi|221079308|gb|EU982038.1| Aspergillus fumigatus strain ATHUM 5013 28S large subunit ribosomal RNA gene and intergenic spacer region partial sequence
Aspergillus fumigatus var. niveus 28S large subunit ribosomal RNA gene and intergenic spacer region
gi|221079307|gb|EU982037.1| Aspergillus flavus strain ATHUM 5033 28S large subunit ribosomal RNA gene and intergenic spacer region partial sequence
gi|221079306|gb|EU982036.1| Aspergillus flavus strain ATHUM 5015 28S large subunit ribosomal RNA gene and intergenic spacer region partial sequence
gi|221079304|gb|EU982034.1| Aspergillus niger strain ATHUM 2539 28S large subunit ribosomal RNA gene and intergenic spacer region partial sequence
gi|221079305|gb|EU982035.1| Aspergillus awamori strain ATHUM 5181 28S large subunit ribosomal RNA gene and intergenic spacer region partial sequence
gi|221079303|gb|EU982033.1| Aspergillus niger strain ATHUM 5044 28S large subunit ribosomal RNA gene and intergenic spacer region partial sequence
gi|221079311|gb|EU982041.1| Neosartorya fischeri strain ATHUM 5030 28S large subunit ribosomal RNA gene and intergenic spacer region partial sequence
gi|221079310|gb|EU982040.1| Aspergillus clavatus strain ATHUM 5036 IGS and 28S large subunit ribosomal RNA (28S) genes and intergenic spacer region partial sequence
gi|221079309|gb|EU982039.1| Aspergillus clavatus strain ATHUM 5032 28S large subunit ribosomal RNA gene and intergenic spacer region partial sequence
gi|221079320|gb|EU982050.1| Aspergillus niveus strain ATHUM 5029 28S large subunit ribosomal RNA gene and intergenic spacer region partial sequence
gi|221079321|gb|EU982051.1| Aspergillus terreus strain ATHUM 4761 28S large subunit ribosomal RNA gene and intergenic spacer region partial sequence
gi|221079317|gb|EU982047.1| Aspergillus oryzae strain ATHUM 4958 28S large subunit ribosomal RNA gene and intergenic spacer region partial sequence
gi|221079316|gb|EU982046.1| Aspergillus parasiticus strain ATHUM 5038 28S large subunit ribosomal RNA gene and intergenic spacer region partial sequence
gi|221079315|gb|EU982045.1| Aspergillus parasiticus strain ATHUM 5037 28S large subunit ribosomal RNA gene and intergenic spacer region partial sequence
gi|221079313|gb|EU982043.1| Aspergillus rubrum strain ATHUM 5183 28S large subunit ribosomal RNA gene and intergenic spacer region partial sequence
gi|221079312|gb|EU982042.1| Eurotium amstelodami strain ATHUM 5082 28S large subunit ribosomal RNA gene and intergenic spacer region partial sequence
gi|221079333|gb|EU982063.1| Aspergillus ochraceus strain ATHUM 5014 28S large subunit ribosomal RNA gene and intergenic spacer region partial sequence
gi|221079336|gb|EU982066.1| Emericella nidulans strain ATHUM 5022 28S large subunit ribosomal RNA gene and intergenic spacer region partial sequence
gi|221079335|gb|EU982065.1| Emericella variecolor strain ATHUM 5019 28S large subunit ribosomal RNA gene and intergenic spacer region partial sequence
gi|221079314|gb|EU982044.1| Aspergillus puniceus strain ATHUM 5434 28S large subunit ribosomal RNA gene and intergenic spacer region partial sequence
gi|221079325|gb|EU982055.1| Aspergillus sydowii strain ATHUM 5093 28S large subunit ribosomal RNA gene and intergenic spacer region partial sequence
gi|221079327|gb|EU982057.1| Aspergillus ustus strain ATHUM 5103 28S large subunit ribosomal RNA gene and intergenic spacer region partial sequence
gi|221079326|gb|EU982056.1| Aspergillus ustus strain ATHUM 5097 28S large subunit ribosomal RNA gene and intergenic spacer region partial sequence
gi|221079332|gb|EU982062.1| Aspergillus aculeatus strain ATHUM 5028 28S large subunit ribosomal RNA gene and intergenic spacer region partial sequence

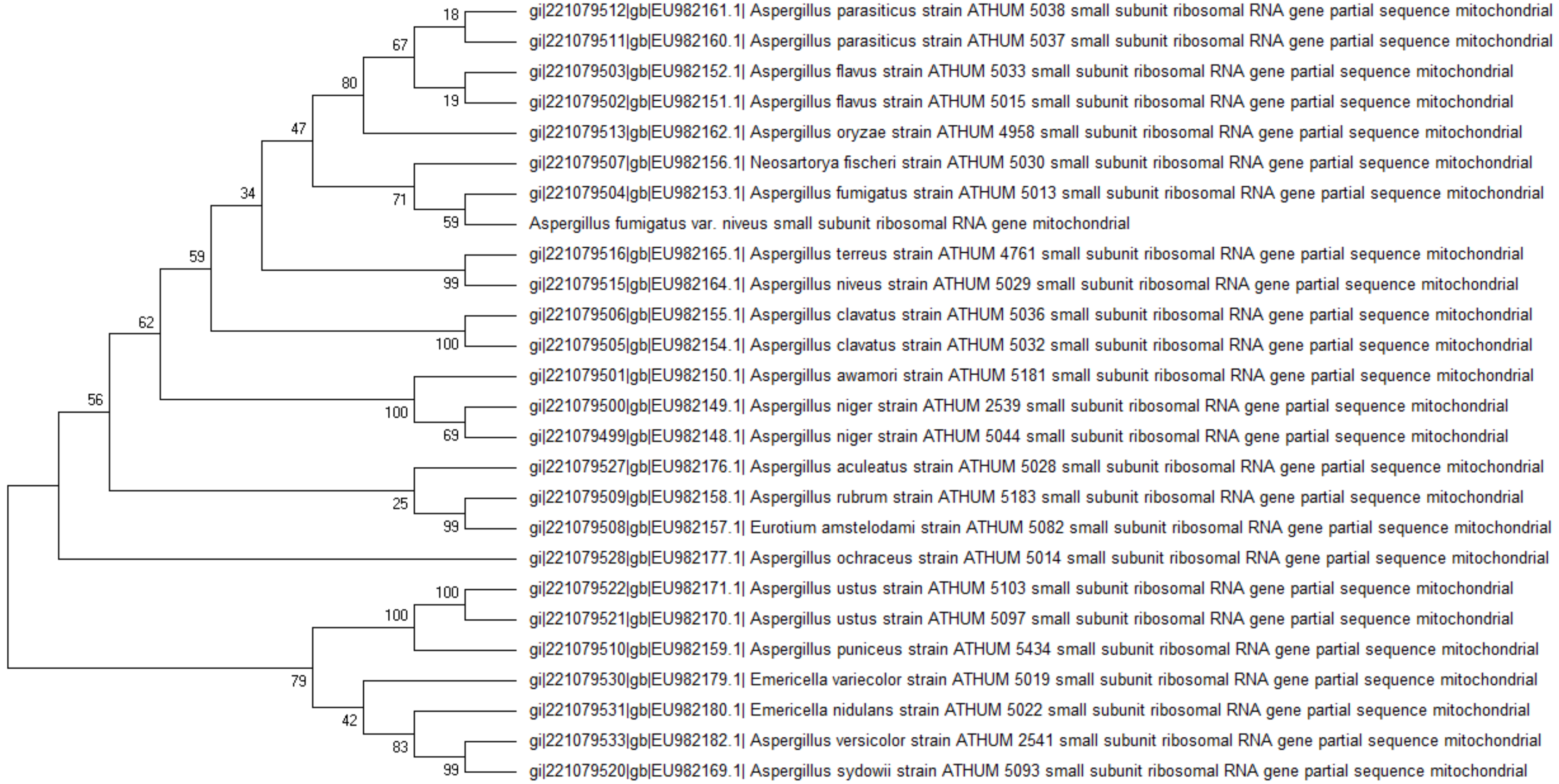

gi|221079512|gb|EU982161.1| Aspergillus parasiticus strain ATHUM 5038 small subunit ribosomal RNA gene partial sequence mitochondrial
gi|221079511|gb|EU982160.1| Aspergillus parasiticus strain ATHUM 5037 small subunit ribosomal RNA gene partial sequence mitochondrial
gi|221079503|gb|EU982152.1| Aspergillus flavus strain ATHUM 5033 small subunit ribosomal RNA gene partial sequence mitochondrial
gi|221079502|gb|EU982151.1| Aspergillus flavus strain ATHUM 5015 small subunit ribosomal RNA gene partial sequence mitochondrial
gi|221079513|gb|EU982162.1| Aspergillus oryzae strain ATHUM 4958 small subunit ribosomal RNA gene partial sequence mitochondrial
gi|221079507|gb|EU982156.1| Neosartorya fischeri strain ATHUM 5030 small subunit ribosomal RNA gene partial sequence mitochondrial
gi|221079504|gb|EU982153.1| Aspergillus fumigatus strain ATHUM 5013 small subunit ribosomal RNA gene partial sequence mitochondrial
Aspergillus fumigatus var. niveus small subunit ribosomal RNA gene mitochondrial
gi|221079516|gb|EU982165.1| Aspergillus terreus strain ATHUM 4761 small subunit ribosomal RNA gene partial sequence mitochondrial
gi|221079515|gb|EU982164.1| Aspergillus niveus strain ATHUM 5029 small subunit ribosomal RNA gene partial sequence mitochondrial
gi|221079506|gb|EU982155.1| Aspergillus clavatus strain ATHUM 5036 small subunit ribosomal RNA gene partial sequence mitochondrial
gi|221079505|gb|EU982154.1| Aspergillus clavatus strain ATHUM 5032 small subunit ribosomal RNA gene partial sequence mitochondrial
gi|221079501|gb|EU982150.1| Aspergillus awamori strain ATHUM 5181 small subunit ribosomal RNA gene partial sequence mitochondrial
gi|221079500|gb|EU982149.1| Aspergillus niger strain ATHUM 2539 small subunit ribosomal RNA gene partial sequence mitochondrial
gi|221079499|gb|EU982148.1| Aspergillus niger strain ATHUM 5044 small subunit ribosomal RNA gene partial sequence mitochondrial
gi|221079527|gb|EU982176.1| Aspergillus aculeatus strain ATHUM 5028 small subunit ribosomal RNA gene partial sequence mitochondrial
gi|221079509|gb|EU982158.1| Aspergillus rubrum strain ATHUM 5183 small subunit ribosomal RNA gene partial sequence mitochondrial
gi|221079508|gb|EU982157.1| Eurotium amstelodami strain ATHUM 5082 small subunit ribosomal RNA gene partial sequence mitochondrial
gi|221079528|gb|EU982177.1| Aspergillus ochraceus strain ATHUM 5014 small subunit ribosomal RNA gene partial sequence mitochondrial
gi|221079522|gb|EU982171.1| Aspergillus ustus strain ATHUM 5103 small subunit ribosomal RNA gene partial sequence mitochondrial
gi|221079521|gb|EU982170.1| Aspergillus ustus strain ATHUM 5097 small subunit ribosomal RNA gene partial sequence mitochondrial
gi|221079510|gb|EU982159.1| Aspergillus puniceus strain ATHUM 5434 small subunit ribosomal RNA gene partial sequence mitochondrial
gi|221079530|gb|EU982179.1| Emericella variecolor strain ATHUM 5019 small subunit ribosomal RNA gene partial sequence mitochondrial
gi|221079531|gb|EU982180.1| Emericella nidulans strain ATHUM 5022 small subunit ribosomal RNA gene partial sequence mitochondrial
gi|221079533|gb|EU982182.1| Aspergillus versicolor strain ATHUM 2541 small subunit ribosomal RNA gene partial sequence mitochondrial
gi|221079520|gb|EU982169.1| Aspergillus sydowii strain ATHUM 5093 small subunit ribosomal RNA gene partial sequence mitochondrial
18
67
80
19
47
71
59
34
59
99
62
100
56
100
69
25
99
100
100
79
42
83
99

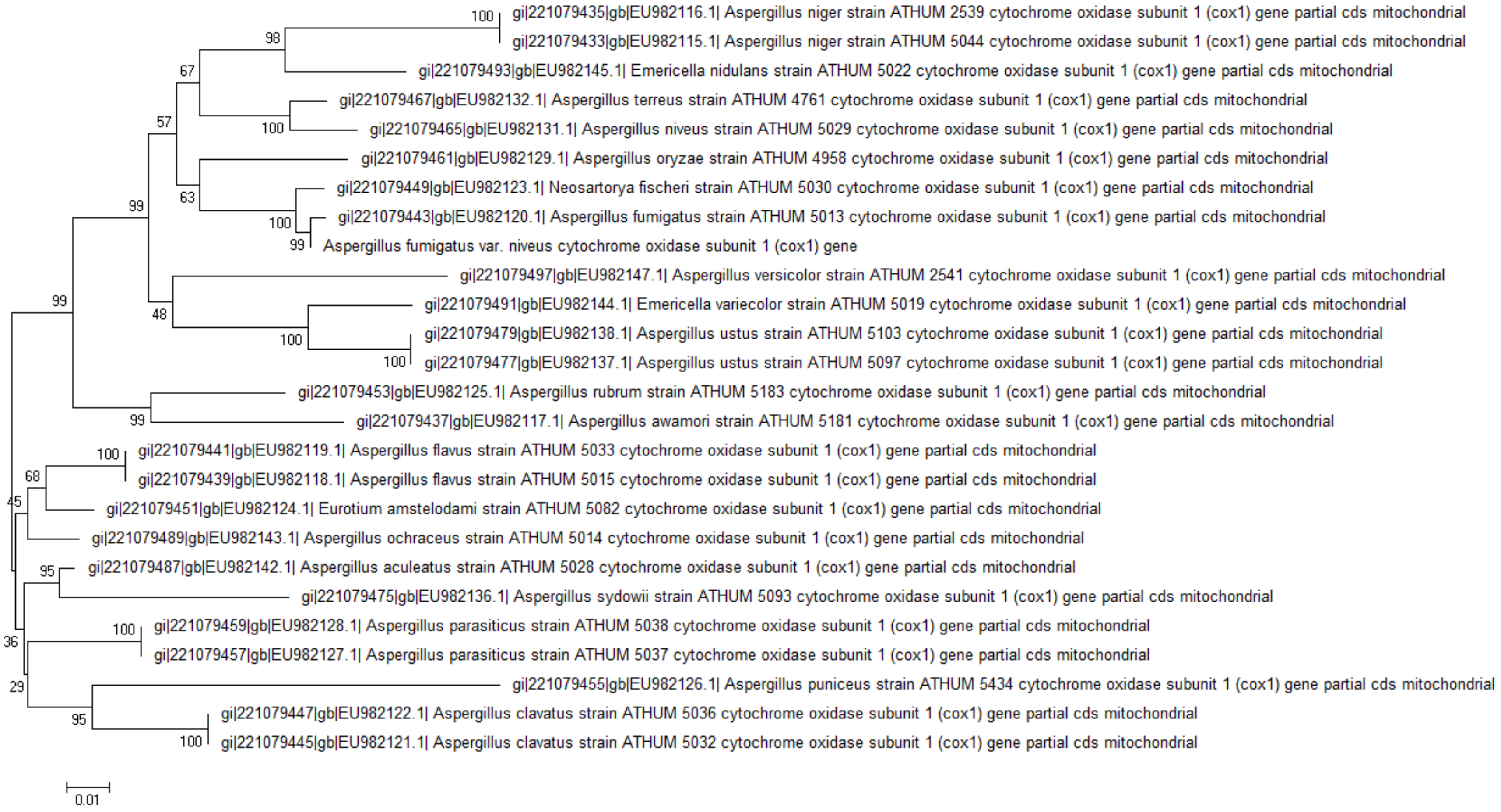
gi|221079435|gb|EU982116.1| Aspergillus niger strain ATHUM 2539 cytochrome oxidase subunit 1 (cox1) gene partial cds mitochondrial
gi|221079433|gb|EU982115.1| Aspergillus niger strain ATHUM 5044 cytochrome oxidase subunit 1 (cox1) gene partial cds mitochondrial
gi|221079493|gb|EU982145.1| Emericella nidulans strain ATHUM 5022 cytochrome oxidase subunit 1 (cox1) gene partial cds mitochondrial
gi|221079467|gb|EU982132.1| Aspergillus terreus strain ATHUM 4761 cytochrome oxidase subunit 1 (cox1) gene partial cds mitochondrial
gi|221079465|gb|EU982131.1| Aspergillus niveus strain ATHUM 5029 cytochrome oxidase subunit 1 (cox1) gene partial cds mitochondrial
gi|221079461|gb|EU982129.1| Aspergillus oryzae strain ATHUM 4958 cytochrome oxidase subunit 1 (cox1) gene partial cds mitochondrial
gi|221079449|gb|EU982123.1| Neosartorya fischeri strain ATHUM 5030 cytochrome oxidase subunit 1 (cox1) gene partial cds mitochondrial
gi|221079443|gb|EU982120.1| Aspergillus fumigatus strain ATHUM 5013 cytochrome oxidase subunit 1 (cox1) gene partial cds mitochondrial
Aspergillus fumigatus var. niveus cytochrome oxidase subunit 1 (cox1) gene
gi|221079497|gb|EU982147.1| Aspergillus versicolor strain ATHUM 2541 cytochrome oxidase subunit 1 (cox1) gene partial cds mitochondrial
gi|221079491|gb|EU982144.1| Emericella variecolor strain ATHUM 5019 cytochrome oxidase subunit 1 (cox1) gene partial cds mitochondrial
gi|221079479|gb|EU982138.1| Aspergillus ustus strain ATHUM 5103 cytochrome oxidase subunit 1 (cox1) gene partial cds mitochondrial
gi|221079477|gb|EU982137.1| Aspergillus ustus strain ATHUM 5097 cytochrome oxidase subunit 1 (cox1) gene partial cds mitochondrial
gi|221079453|gb|EU982125.1| Aspergillus rubrum strain ATHUM 5183 cytochrome oxidase subunit 1 (cox1) gene partial cds mitochondrial
gi|221079437|gb|EU982117.1| Aspergillus awamori strain ATHUM 5181 cytochrome oxidase subunit 1 (cox1) gene partial cds mitochondrial
gi|221079441|gb|EU982119.1| Aspergillus flavus strain ATHUM 5033 cytochrome oxidase subunit 1 (cox1) gene partial cds mitochondrial
gi|221079439|gb|EU982118.1| Aspergillus flavus strain ATHUM 5015 cytochrome oxidase subunit 1 (cox1) gene partial cds mitochondrial
gi|221079451|gb|EU982124.1| Eurotium amstelodami strain ATHUM 5082 cytochrome oxidase subunit 1 (cox1) gene partial cds mitochondrial
gi|221079489|gb|EU982143.1| Aspergillus ochraceus strain ATHUM 5014 cytochrome oxidase subunit 1 (cox1) gene partial cds mitochondrial
gi|221079487|gb|EU982142.1| Aspergillus aculeatus strain ATHUM 5028 cytochrome oxidase subunit 1 (cox1) gene partial cds mitochondrial
gi|221079475|gb|EU982136.1| Aspergillus sydowii strain ATHUM 5093 cytochrome oxidase subunit 1 (cox1) gene partial cds mitochondrial
gi|221079459|gb|EU982128.1| Aspergillus parasiticus strain ATHUM 5038 cytochrome oxidase subunit 1 (cox1) gene partial cds mitochondrial
gi|221079457|gb|EU982127.1| Aspergillus parasiticus strain ATHUM 5037 cytochrome oxidase subunit 1 (cox1) gene partial cds mitochondrial
gi|221079455|gb|EU982126.1| Aspergillus puniceus strain ATHUM 5434 cytochrome oxidase subunit 1 (cox1) gene partial cds mitochondrial
gi|221079447|gb|EU982122.1| Aspergillus clavatus strain ATHUM 5036 cytochrome oxidase subunit 1 (cox1) gene partial cds mitochondrial
gi|221079445|gb|EU982121.1| Aspergillus clavatus strain ATHUM 5032 cytochrome oxidase subunit 1 (cox1) gene partial cds mitochondrial
0.01
